# Take the sex out of STI screening! Views of young women on implementing chlamydia screening in General Practice

**DOI:** 10.1186/1471-2334-8-62

**Published:** 2008-05-09

**Authors:** Natasha L Pavlin, Rhian Parker, Christopher K Fairley, Jane M Gunn, Jane Hocking

**Affiliations:** 1 Department of General Practice, University of Melbourne, Victoria, Australia; 2 Healthpact Research Centre for Health Promotion and Wellbeing, University of Canberra, ACT, Australia; 3 Department of Public Health, University of Melbourne, Victoria, Australia; Melbourne Sexual Health Centre, 580 Swanston St, Melbourne, Victoria, Australia; 4 Key Centre for Women's Health in Society, University of Melbourne, Victoria, Australia

## Abstract

**Background:**

Australia is developing a chlamydia screening program. This study aimed to determine the attitudes of young women to the introduction of chlamydia screening in Australian General Practice.

**Methods:**

In-depth face-to-face interviews with 24 young women from across Victoria, Australia, attending a randomly selected sample of general practices.

**Results:**

Young women reported that they would accept age-based screening for chlamydia in general practice, during both sexual-health and non-sexual-health related consultations. Trust in their general practitioner (GP) was reported to be a major factor in the acceptability of chlamydia screening. The women felt chlamydia screening should be offered to *all *young women rather than targeted at "high risk" women based on sexual history and they particularly emphasised the importance of normalising chlamydia screening. The women reported that they did not want to be asked to provide a sexual history as part of being asked to have a chlamydia test. Some reported that they would lie if asked how many partners they had had

**Conclusion:**

Women do not want a sexual history taken when being asked to have a chlamydia test while attending a general practitioner. They prefer the offer of chlamydia screening to be based on age rather than assessment of sexual risk. Chlamydia screening needs to be normalised and destigmatised.

## Background

*Chlamydia trachomatis *infection is the most commonly notified sexually transmitted infection in Australia and is most prevalent in people under the age of 25 [1]. In women, chlamydia infection is usually asymptomatic, can persist for months to years and result in pelvic inflammatory disease and infertility[2]. Chlamydia infection in men is more often symptomatic and is not thought to have such direct adverse health consequences. Chlamydia screening is currently thought to be of most benefit in sexually active women under the age of 25 [3]. Chlamydia screening programs exist in Sweden [4] and the United Kingdom [5] with widespread testing also occurring in the Netherlands [6] and Denmark [6]. Australia has allocated 12.5 million dollars to determine the optimal national approach to introducing chlamydia screening. Most countries undertake chlamydia screening, at least in part, through primary health care.

Australia, like the UK, has a network of general practitioners (GPs) that would be ideally placed to conduct widespread chlamydia screening. Nearly 90% of Australian women aged 15–24 years visit a GP at least once each year [7]. Currently only about 7% of these are tested for chlamydia [8].

Successful screening programs require support from the target group to be screened; thus target individual's views on chlamydia screening, and in particular any perceived adverse consequences, are important in screening program design so that participation can be maximised [9].

This study aimed to determine how Australian young women feel about being asked to test for chlamydia when they attend a GP for any reason; the potential psychosocial impacts of chlamydia screening and to determine what information and support young women would find helpful.

## Methods

The study was conducted in the State of Victoria, Australia (population about five million). Ethics approval was obtained from Melbourne University Human Research Ethics Committee. All participants gave informed consent. We conducted in-depth, face-to-face semi-structured interviews with 24 women aged 16 to 25 between November 2005 and February 2006. The interview schedule was devised with input from all members of our multi-disciplinary research team and was informed by current knowledge about chlamydia screening and young women's attitudes to screening [9] and by our research questions (see Additional file 1). We were careful to balance individual or discipline bias in assembling our team: an experienced sexual health and infectious diseases physician, two practising GPs with academic experience, a sociologist with expertise in qualitative research and an epidemiologist and biostatistician with a particular interest in chlamydia infection. All have useful experience in relation to chlamydia screening in Australia and their different backgrounds helped to ensure a broad range of perspectives informed the study. The interview schedule was piloted with two young women and amended following review by RP and NP. We intentionally chose NP as the interviewer as she is a young woman able to relate to the interviewees and engender their trust and thus improve the quality of responses and also as she has experience dealing with sensitive issues in her role as a GP. NP conducted all interviews and was supervised in this by RP who regularly listened to interview recordings and discussed them with NP. RP has extensive experience conducting interviews on sensitive topics and has acknowledged skills in qualitative research.

Women were eligible if they were sexually active, aged 16 to 25 and attended a GP participating in the Victorian GP training network. To obtain a reasonable representation of women from different geographical locations in Victoria, GPs were selected according to the Rural, Remote and Metropolitan Areas (RRMA) classification of their primary practice. Of the 30 GPs approached, 27 agreed to be potential recruitment sites. Over two consecutive consulting days GPs were asked to seek the consent of eligible women to be contacted by a researcher to discuss the study.

Interviews were conducted initially with women from metropolitan areas of Victoria (RRMA 1). After the first eight interviews it became apparent we were reaching data saturation (common themes evident in the interview data with no new themes arising). Interviews with rural and regional women continued until we had eight interviews from each geographic area.

Interviews were transcribed verbatim and imported into NVivo7 as Word documents. We applied a thematic analysis and looked for emerging themes [10]. NP and RP read all transcripts and met to consider possible themes. The coding framework developed from these discussions. NP and RP reviewed emerging themes as the analysis progressed bringing their different perspectives: GP researcher and sociologist. We attempted to avoid bias in interpreting the data by having both RP and NP active in reviewing the transcripts, eliciting themes and formulating our analysis. The broader research team also met regularly to discuss the findings and challenge them from various angles thus bringing the benefit of our broad range of backgrounds. We were satisfied we had reached data saturation as no new themes appeared after the initial eight interviews although a further 16 were completed.

## Results

Over the two study days 51 eligible women were seen by the GPs, 45 were asked to participate, and 36 agreed. Of these, interviews were undertaken with 24 (66%). Reasons for failure to be interviewed included: unable to contact the woman (1), unable to find a suitable time for the interview (1), refusal to be interviewed once contacted (1) and the decision on the part of the research team not to arrange interviews due to data saturation (9). The women were evenly distributed between urban, regional and rural areas. They were primarily recruited by female GPs.

### How women thought screening could work

The women felt *how *they were approached about having a chlamydia test was important and that comprehensive information about chlamydia should be provided when the test was offered. There was strong agreement that age-based screening, for instance, of young women between 16 and 24 years, would be acceptable and non-discriminatory:

*So I think you have to make it known, just even if they come out and say 'look, we screen everybody in this age and you're in that category...and we'd basically like to do that on you just to make sure.' And I wouldn't even go into detail or ask them how many people they've slept with*.

Introducing the issue of chlamydia screening during a sexual health-related consultation was seen as appropriate as it was directly related to the consultation:

*I think it depends on the circumstances of why I'm there. Like I said, if I was just purely going for a Pap smear, well then, you know, you're showing everything anyway*.

### Need to normalise chlamydia

Normalising chlamydia was seen as important to minimise stigma associated with chlamydia infection:

*Well, Pap smears have been normalised. Community awareness, um, screening programs for everyone*.

The women felt that chlamydia should be framed as a public health issue that is openly discussed rather than an issue that relates to the behaviour of an individual:

*If there was something in the waiting-room that said your doctor may ask you about having the test, then you're a little bit more prepared for it, and if you saw in the paper that it was becoming a big issue and they'd probably ask you about it next time you go in...So it's much more a public health issue than a individual, yeah, hush-hush kind of*....

A widespread community education campaign was seen as a crucial factor in normalising chlamydia testing and diagnosis.

### Sexual history taking before a chlamydia test

These young women emphasised there should be no pressure put on women to provide a sexual history. Some felt that questions relating to number of partners would be a barrier to accepting a chlamydia test and that they would not answer them truthfully in any case:

*Yeah. I wouldn't, I think I would lie about it if I got asked*.

Young women were concerned about being judged and may not disclose sexual activity because of this:

*No, that's something that would shock me, like, that's something that you shouldn't be asking*.

### Psychosocial implications of chlamydia screening

Concerns were raised about the confronting nature of being tested for chlamydia and about fear of the infection:

*..cause it's, like, you know, a scary word, and especially young girls it would probably just...I know it would scare the crap out of me*.

There was particular concern that young women diagnosed with chlamydia would be judged and seen as sexually promiscuous:

*There's a big stigma if you get something and that makes it really hard for people to get tested 'cause you feel like you're dirty or you're worthless or you've done something wrong*.

However, although some young women felt that they would be '*embarrassed and ashamed' *if they were diagnosed with chlamydia, others commented that the initial shock would turn to relief that the infection had been detected and could be treated:

...like, it's not anything that anyone likes to hear, but I think it's good because it does give you peace of mind and you're, like, 'oh, at least now I know. I can do something about it.'

### Management of chlamydia infection

Women were asked about how they would want to receive their chlamydia test results. When asked, the young women were clear that they did not want to be contacted by SMS (mobile text messaging):

*... some people might go through your phone and read the message or whatever*.

Some women felt being contacted by email would be acceptable but a number did not have access to a computer and many had privacy concerns about email (although not as much as for SMS):

*I don't like the Internet so much because, you know, anyone could view that, anyone could get in. You know it could be your little brother gets in and finds your results*.

Many women felt receiving a letter to their home address would be acceptable, both for results and for recalls, as long as it was in an unmarked envelope. A phone-call was acceptable (particularly to mobile phones) but face-to-face with their GP was definitely preferred for receiving results, especially if positive.

*A phone call would be best to say to come in, um, 'we need to talk to you about your test results,' and definitely one on one*.

### Partner notification

Interestingly more than half of the women interviewed thought that they probably would not tell their partners, particularly if it was a more casual sexual contact, or if they had subsequently broken up with that person:

I don't think I would tell somebody I wasn't with um, for a long period, that I didn't, wasn't in an official relationship with, because you'd think, you'd probably think, 'that's my business, not theirs.'

Some of the reasons given for not telling partners included being fearful of negative gossip and worrying about what would be the reactions of partners if they were told they might have been exposed to chlamydia:

*...you could be, you know, down at the footy club or wherever and blah, blah, blah and there you go, you're known as the town bike...you see, I initially came from a small town, so I know gossip travels very quickly, so that kind of sticks in the back of my mind*.

Most women thought they would feel very uncomfortable about telling partners that they may have been exposed to chlamydia. Those women who thought they would notify partners were more likely to be older and to be in a stable relationship. A feeling of responsibility toward their partners and thinking chlamydia is a serious disease was associated with being more likely to tell partners.

Almost all the women interviewed thought that anonymous contact tracing would be useful and would make it easier to notify partners who would otherwise not be contacted. However, some women saw the idea of anonymous notification as cowardly and disrespectful.

## Discussion

This is one of the first qualitative studies to investigate women's attitudes to chlamydia screening in general practice [11,12]. Other studies have assessed women within a sexual health or family planning clinic setting [11,13-15] and have not reported as much concern from women about being asked their sexual history [11,13-15]. We were particularly interested to learn that women in a general practice setting did not like being asked to provide a sexual history to their GP. This finding has not been published elsewhere and is worthy of comment. There is considerable evidence suggesting that GPs see having to take a sexual history as a barrier to STI testing in general practice [16,17]. This raises the question – is it necessary to take a sexual history when screening for chlamydia?

We think our finding that women are concerned about providing a sexual history highlights an important difference between screening for chlamydia in general practice versus screening in a sexual health clinic: namely, offering a chlamydia test in a sexual health or family planning clinic is automatically put in context by the nature of the environment, whereas in general practice the offer may seem to come "out of the blue". The importance of normalising the offer of chlamydia testing, so that individual women do not feel singled out, cannot be overemphasized

We found reasonable consensus among the women interviewed that it was acceptable to suggest testing based solely on age or when they presented for a sexual health-related consultation. Screening on the basis of risk factors such as number of sexual partners would not work because several women stated that they would lie about their number of sexual partners if asked by their GP. This finding is not surprising as discrepancies between men and women in sexual partner reporting are widely acknowledged, with the number of female partners reported by men exceeding the number of male partners reported by women [18-20].

The women we interviewed accepted the idea of partner-notification as a necessary aspect of chlamydia screening, but varied in their personal commitment to telling partners if they were diagnosed with chlamydia. Ensuring women have access to anonymous partner-notification services and also good support from their GP may improve this process. It also seems important to acknowledge that the women interviewed found neither email nor SMS acceptable for results/partner-notification. In other countries, notably the UK, these forms of "new technology" are already in widespread use in chlamydia screening. If SMS/email contact systems are planned to be part of an Australian program, more research is needed to understand how they can best be used in the Australian context.

One of the limitations of our study is that the women interviewed were primarily recruited by female GPs and our sample size was relatively small. Also the women interviewed had not necessarily undergone chlamydia screening and their opinions are not informed by personal experience. The structure of our study was biased against including the voices of socially disadvantaged women as the women were required to be contactable and to organise appointments in advance and this was difficult for those with a more chaotic lifestyle. Strengths include our inclusion of women from rural, regional and urban Australia and that data saturation was reached very quickly. It is worth noting that the sentiments expressed by our interviewees are similar to those in other qualitative work looking at the views of young people diagnosed with chlamydia [21]. At the time our study was designed, Australian policy accepted the idea that chlamydia screening should be aimed primarily at women, as they experience the most serious effects of chlamydia infection; hence our study was focused on the views of young women. It is increasingly recognised that infections in men are also an important aspect of chlamydia control and men's views are necessarily of interest. This may be an area for further research.

## Conclusion

Normalising chlamydia testing and diagnosis is seen as important if screening is to be successful. Young women report that being required to disclose their sexual history is a barrier to their accepting chlamydia screening. Chlamydia *is *an STI and notification and treatment of sexual partners *is *important. Understanding this promotes young women's acceptance of chlamydia screening. However, is a *detailed *sexual history really an important precursor to a chlamydia test? Our study suggests that this question warrants further exploration.

## Competing interests

The authors declare that they have no competing interests.

## Authors' contributions

JH was the PI. NLP conducted the interviews and data analysis and was the lead author for this paper. RP supervised the conduct of the interviews and contributed to the data analysis. All authors contributed to study design and the preparation of the manuscript.

## Pre-publication history

The pre-publication history for this paper can be accessed here:



## Supplementary Material

Additional file 1 Interview schedule. The interview schedule used to guide the semi-structured interviews with young women on their views about chlamydia testing in general practice. Click here for file
