# Supplementary material for: Take the sex out of STI screening! Views of young women on implementing chlamydia screening in General Practice
Source: BMC Infect Dis. 2008 May 9;8:62. doi: 10.1186/1471-2334-8-62 (PMC2394525; doi:10.1186/1471-2334-8-62)
Supplement: Additional file 1 — Interview schedule. The interview schedule used to guide the semi-structured interviews with young women on their views about chlamydia testing in general practice. [file 1471-2334-8-62-S1.pdf]

Table 1. Interview schedule

**Objectives**
To determine what young women in the target age group (16 – 24) know about chlamydia and how they feel about the idea of being offered chlamydia screening. How would these young women feel about being asked to test for chlamydia when they attend a GP for any reason? What psychosocial issues do young women think might arise for them from chlamydia screening? What level of information do these young women feel they require about chlamydia screening and what form would that information best come in?

---

**Preamble**
Introduce researcher
Outline objectives of research
Describing/demographic questions

1. DOB
2. Living Situation – with family/independent/partnered/children
3. Work – Full/part time
4. Study/school

---

1.Have you heard of chlamydia?
If no
Standardised Chlamydia information provided:
Key points – something you can get from having sex; often no symptoms or warning signs; very common; easy to test for (urine test – non-invasive); easy to treat but if not treated can be quite serious. Can cause pelvic pain that is difficult to treat and infertility.
Need to stress it is really really common
If yes
2.What do you know about it?
If lacking any information or have incorrect information
Standardised Chlamydia information provided:
Key points – something you can get from having sex; often no symptoms or warning signs; very common; easy to test for (urine test – non-invasive); easy to treat but if not treated can be quite serious

---

3. What do you know about screening tests for chlamydia?
Information on screening provided – test instigated by GP, no symptoms of disease. For chlamydia – urine or swab test. Probably at least once a year, maybe once every 6 months for sexually active young women.

---

How would you feel if your GP recommended that you have a chlamydia test?
Explore responses
Probes- what if in conjunction with consult related to sexual/reproductive health?

- what if asked at any consult?
- - what if there were a recall/reminder system eg a letter in the mail, sms, email?
- ? different depending on GP? – what characteristics important? GP known for longer (perhaps more trust) /new GP (perhaps seen as more confidential)?
- What about self-testing eg a test you could buy at the pharmacy and post in?

---

5. What do you think other young women like you need to be told about chlamydia and chlamydia screening?

---

6.Where would you like to access this information?
Probes - Internet? Schools? Youth Groups? Written information?- pamphlets, letters, TV ads, Billboards, GP? Nurses? Community Health Centre? Sexual Health Centre? Sexual Health Forum? Peer educators? Website? Hotline?

---

7. If you were tested for chlamydia by your GP and found you had chlamydia how would you feel?
What support would you need? Who from? What support would you need from your GP?
How would you like to get the test results? – another GP visit, letter, email, SMS message etc

---

8.How would you feel about telling your sexual partner(s)?

---

Current treatment for chlamydia involves taking 2 antibiotic tablets which stay active in your system for a week. Ideally any people you had had sex with over the last 3 months or so should also be treated.
9. What do you think about current chlamydia treatment?
Discuss partner notification, follow up testing etc.

---

Anything else you would like to say?
Thank you for your time
